# Persistence with anti-tumour necrosis factor therapies in patients with psoriatic arthritis: observational study from the British Society of Rheumatology Biologics Register

**DOI:** 10.1186/ar2670

**Published:** 2009-04-08

**Authors:** Amr A Saad, Darren M Ashcroft, Kath D Watson, Kimme L Hyrich, Peter R Noyce, Deborah PM Symmons, the British Society for Rheumatology Biologics Register

**Affiliations:** 1 School of Pharmacy and Pharmaceutical Sciences, University of Manchester, Oxford Road, Manchester M13 9PT, UK; 2 Arthritis Research Campaign Epidemiology Unit, University of Manchester, Oxford Road, Manchester M13 9PT, UK

## Abstract

**Introduction:**

Anti-TNF therapies represent a breakthrough in the treatment of severe psoriatic arthritis. However, little is known about long-term drug persistence with these treatments in patients with psoriatic arthritis in routine clinical practice. The aim of this study was to assess persistence with first-course and second-course treatment with anti-TNF agents in a prospective cohort of psoriatic arthritis patients and to identify factors associated with and reasons for drug discontinuation.

**Methods:**

A total of 566 patients with psoriatic arthritis were registered with the British Society for Rheumatology Biologics Register (first anti-TNF agent: etanercept, n = 316; infliximab, n = 162; and adalimumab, n = 88). Treating physicians completed 6-monthly follow-up questionnaires detailing changes to anti-TNF therapies. Persistence with treatment was examined using Kaplan–Meier survival analysis. Reasons for withdrawal were classified as due to inefficacy, adverse events or other reasons. Univariate and multivariate Cox proportional hazard models were developed to examine potential predictors of withdrawals due to inefficacy or adverse events, using a range of demographic, baseline disease-specific and therapeutic variables.

**Results:**

At baseline, the mean (standard deviation) age of patients was 45.7 (11.1) years, 53% were female and the mean disease duration was 12.4 (8.7) years. Persistence data were available for a mean (standard deviation) follow-up of 2.3 (0.9) person-years. In total, 422 patients had completed at least 12 months of follow-up, 75.5% of whom remained on their first anti-TNF drug while 9.5% discontinued due to inefficacy, 10.0% due to adverse events and 5.0% due to other reasons. During the period of follow-up, 178 patients received a second anti-TNF therapy. The survivor function on second anti-TNF for switchers was 74% at 12 months.

**Conclusions:**

Psoriatic arthritis patients show high persistence rates with both initial and second anti-TNF therapies.

## Introduction

The development of anti-TNF therapies has dramatically improved the management of a range of autoimmune diseases, including psoriatic arthritis (PsA). TNF acts in the early stages of the inflammatory process, during which it can stimulate T-cell activation and induce the expression of IL-2, IFNγ receptors, proinflammatory cytokines (such as IL-1 and IL-12) and proinflammatory chemokines (such as IL-8) [1].

The currently available anti-TNF agents (etanercept, infliximab and adalimumab) have been studied in a number of randomised controlled trials that assessed their efficacy and safety in PsA [2-7]. A recent meta-analysis of these trials reported substantial improvements (versus placebo) in the signs and symptoms of PsA following anti-TNF therapy [8]. Although there have been no direct head-to-head comparisons of anti-TNF therapies, indirect analysis of the randomised controlled trial data suggested that the three agents were similar in terms of efficacy and safety following short-term use (up to 24 weeks) [8].

There are only limited data available examining long-term persistence with these therapies in patients with PsA who are managed in routine clinical practice [9]. The present study was undertaken to explore persistence with anti-TNF therapies in a large, prospective, population-based cohort of patients with PsA. Specifically, we aimed to evaluate persistence in the use of the first and second anti-TNF therapy, and to identify potential predictors of drug discontinuation and reasons for withdrawal due to adverse events.

## Materials and methods

Subjects included in the study were selected from the British Society for Rheumatology Biologics Register (BSRBR) [10]. The BSRBR aims to recruit patients with rheumatic diseases receiving anti-TNF therapies in the UK. Although primarily a register of patients with rheumatoid arthritis (RA), the BSRBR has also collected data on patients with other rheumatic diseases, as diagnosed by a rheumatologist, who have started therapy with an anti-TNF agent. The register does not have any exclusion criteria other than patients must be registered within 6 months of starting therapy. From 2002 to 2006, the BSRBR recruited patients starting anti-TNF therapies for PsA. The current analysis was restricted to subjects with a physician diagnosis of PsA who had started treatment with etanercept, infliximab or adalimumab.

The British Society for Rheumatology guidelines for PsA published in February 2005 recommend that anti-TNF drugs should be reserved for patients with active PsA (defined as ≥ 3 tender joints and ≥ 3 swollen joints) despite adequate therapeutic trials of at least two standard disease modifying anti-rheumatic drugs individually or in combination [11]. During the study, etanercept (licensed in 2002) was administered as a subcutaneous injection of 25 mg twice weekly or 50 mg once weekly; adalimumab (licensed in 2005) was administered as a subcutaneous injection of 40 mg every 2 weeks. In 2004, infliximab was licensed for use in the management of psoriatic arthritis at a recommended dose of 5 mg/kg administered at weeks 0, 2, 6 and 8, and then every 8 weeks thereafter [12,13]. It is also recommended that infliximab be administered in combination with methotrexate [12].

### Baseline assessment

At the time of initiation of the anti-TNF therapy, the rheumatologist or rheumatology nurse specialist completed a consultant baseline questionnaire that collected data on the patient's age, gender, diagnosis and disease duration, and information about current disease activity, including swollen and tender joint counts (based on the 28-joint count), the erythrocyte sedimentation rate and/or the C-reactive protein level. The 28-joint Disease Activity Score (DAS-28) was then calculated [14]. Details of past and present antirheumatic therapies and current co-morbidities were also recorded.

Each patient completed a patient baseline questionnaire that included details about their current work status, ethnicity, smoking history, the Health Assessment Questionnaire (HAQ) adapted for British use [15], and the Short Form-36 survey [16].

### Follow-up

The rheumatology consultants/nurse specialists were sent a 6-monthly postal follow-up questionnaire for 3 years and then annual follow-ups thereafter. This consultant follow-up questionnaire recorded details of all anti-TNF therapies received, including start and stop dates and reasons for discontinuation. The reasons for stopping were documented by the clinician and were classified into lack of efficacy, adverse events (AEs), or other reasons. In addition, data were recorded for calculation of the DAS-28. Details of all AEs resulting in drug discontinuation were classified using the MedDRA system organ classification [17].

### Data analysis

Analysis was limited to those patients for whom at least one consultant follow-up questionnaire had been returned, as data on drug persistence were otherwise not available. Persistence with anti-TNF therapies was examined for both the first and second treatment courses using Kaplan–Meier survival analysis. For the purpose of this study, drug persistence was defined as 'the length of time from initiation to discontinuation of therapy' [18]. Patients were censored at the date therapy was stopped, the date the patient was switched to another anti-TNF therapy, the date at the end of follow-up or the date the patient died, whichever came first. As patients may often stop treatment during an infection or elective surgery, any gaps in the use of the same anti-TNF treatment of less than 3 months were considered as continuous treatment. Differences in drug persistence between anti-TNF therapies were also examined using Kaplan–Meier survival analysis.

Univariate and multivariate Cox proportional hazard models were used to identify factors associated with drug discontinuation of the first course of anti-TNF therapy. Separate models were developed for overall discontinuation, for discontinuation due to inefficacy and for discontinuation due to adverse events. The following covariates were examined in the models: baseline demographic variables (age (years), therapy start year, gender, current smoking status (yes/no), whether the patient had additional baseline co-morbidities (yes/no)), baseline disease-specific variables (DAS-28 as well as its individual components – high inflammatory markers (C-reactive protein) >20 mg/l and/or erythrocyte sedimentation rate >28 mm/hour – 28-tender joint count and 28-swollen joint count, HAQ, disease duration (years)) and therapeutic variables (anti-TNF therapy used and concurrent use of methotrexate, sulfasalazine or steroids (yes/no)). In the multivariate analyses, we used the composite DAS-28 score as a potential predictor rather than its individual components. The results are presented as hazard ratios (HRs) with corresponding 95% confidence intervals (CIs). All calculations were undertaken using STATA version 9.0 [19].

### Ethical approval

The study was approved by the North West NHS Multicentre Research Ethics Committee, and all subjects gave their written consent for participation.

## Results

### Baseline demographic and disease characteristics

A total of 596 biologically naive PsA patients were registered with the BSRBR. Persistence data were available for 566 patients with PsA (95%). Of these, 316 (55.8%) patients commenced treatment with etanercept, 162 (28.6%) patients started with infliximab and 88 (15.6%) patients started with adalimumab.

The baseline characteristics of the PsA patient cohort and the three anti-TNF groups are presented in Table 1. At baseline, the mean (standard deviation) age of all patients was 45.7 (11.1) years, 53% were female and the mean (standard deviation) disease duration was 12.4 (8.7) years. The median HAQ score was 1.9 (interquartile range 1.4 to 2.3). Similar demographic and disease characteristics were observed for patients included in each of the anti-TNF treatment groups.

**Table 1:** Demographic and disease characteristics of the psoriatic arthritis patient cohort at baseline

Characteristic	Complete psoriatic arthritis cohort (n = 566)	Etanercept (n = 316)	Infliximab (n = 162)	Adalimumab (n = 88)	*P *value
Demographic					
Age (years)	45.7 ± 11.1	45.8 ± 11.1	44.8 ± 11.0	47.0 ± 11.6	0.325
Female	300 (53.0)	162 (51.3)	89 (54.9)	47 (53.4)	0.581
Disease duration (years)	12.4 ± 8.7	12.8 ± 9.0	12.2 ± 8.0	11.4 ± 8.4	0.384
Patients with other baseline co-morbidities	375 (62.9)	191 (57.4%)	117 (68.4%)	67 (72.8%)	0.111
Disease					
28-Tender joint count	13.4 ± 7.7	13.5 ± 7.6	14.1 ± 8.1	12.1 ± 7.1	0.346
28-Swollen joint count	8.9 ± 6.1	8.8 ± 6.1	8.8 ± 6.4	9.7 ± 5.7	0.293
Erythrocyte sedimentation rate (mm)	40.5 ± 29.0	39.4 ± 28.1	44.2 ± 31.4	37.7 ± 27.4	0.459
C-reactive protein level (mg/dl)	39.3 ± 47.1	35.4 ± 41.8	47.8 ± 50.0	35.0 ± 56.6	0.787
28-joint Disease Activity Score	6.4 ± 5.6	6.1 ± 1.2	7.3 ± 10.1	6.0 ± 1.0	0.464
Health Assessment Questionnaire	1.9 (1.4 to 2.3)	1.8 (1.4 to 2.3)	2.0 (1.4 to 2.4)	1.8 (1.1 to 2.3)	0.581

Data presented as the mean ± standard deviation, n (%) or median (interquartile range). *P *value tests for significant differences between the three anti-TNF cohorts.

At baseline, 37.0%, 30.7% and 32.3% of patients had no co-morbidity, one co-morbidity or more than one co-morbidity, respectively. The most frequent co-morbid condition was hypertension, which was present in 29.2% of the patients. Baseline co-morbidities were classified by body systems into cardiovascular (33.9%), pulmonary (13.9%), endocrine (6.7%), gastrointestinal (13.6%) and central nervous system disorders (21.6%). There was no significant difference between the three agents in the presence of baseline co-morbidities.

### Persistence with the first anti-TNF therapy

The mean (standard deviation) follow-up for all patients was 2.3 (0.9) person-years. The mean length of follow-up for patients receiving infliximab was 2.5 person-years, for etanercept was 2.1 person-years and for adalimumab was 1.6 person-years – reflecting the time that these drugs became readily available on the market. Kaplan–Meier survival analyses showing rates of discontinuation for inefficacy or AEs are presented in Table 2. Among the 566 patients, 422 patients completed at least 12 months of follow-up, 75.5% of whom remained on their first anti-TNF therapy while 9.5% discontinued treatment due to inefficacy, 10.0% discontinued due to AEs and 5.0% due to other reasons. The survivor functions for withdrawals due to inefficacy were 0.87 in the second year and 0.80 in the third year of treatment, while that of withdrawals due to AEs was 0.92 and 0.87, respectively (Table 2).

**Table 2:** Survivor function for patients stopping their initial anti-TNF therapy by year of follow-up

Reasons for drug discontinuation	All anti-TNF first course	Etanercept	Infliximab	Adalimumab	All-anti-TNF second course
All reasons					
Year 1	0.82 (0.79 to 0.85)	0.86 (0.81 to 0.89)	0.71 (0.63 to 0.77)	0.91 (0.82 to 0.95)	0.74 (0.71 to 0.78)
Year 2	0.70 (0.66 to 0.74)	0.79 (0.73 to 0.83)	0.52 (0.44 to 0.59)	0.70 (0.54 to 0.81)	0.66 (0.61 to 0.71)
Year 3	0.59 (0.53 to 0.64)	0.65 (0.55 to 0.73)	0.43 (0.35 to 0.51)	0.66 (0.49 to 0.79)	-
Inefficacy					
Year 1	0.92 (0.89 to0.94)	0.94 (0.91 to 0.96)	0.87 (0.81 to 0.92)	0.93 (0.85 to 0.97)	0.70 (0.63 to 0.75)
Year 2	0.87 (0.83 to 0.89)	0.92 (0.88 to 0.94)	0.78 (0.69 to 0.84)	0.80 (0.64 to 0.89)	0.63 (0.55 to 0.69)
Year 3	0.80 (0.75 to 0.85)	0.86 (0.78 to 0.92)	0.79 (0.58 to 0.77)	0.75 (0.57 to 0.87)	-
Adverse events					
Year 1	0.96 (0.94 to 0.97)	0.97 (0.94 to 0.98)	0.93 (0.87 to 0.96)	0.99 (0.92 to 0.99)	0.76 (0.69 to 0.81)
Year 2	0.92 (0.89 to 0.95)	0.95 (0.92 to 0.97)	0.86 (0.78 to 0.91)	0.92 (0.75 to 0.98)	0.64 (0.55 to 0.71)
Year 3	0.87 (0.84 to 0.92)	0.91 (0.84 to 0.95)	0.72 (0.72 to 0.89)	0.92 (0.75 to 0.98)	-

Data presented as mean (95% confidence interval).

Details of AEs leading to withdrawal of treatment for the three anti-TNF agents are presented in Table 3. The leading cause of drug discontinuation for infliximab was infusion reactions (7.4% of patients receiving infliximab, ~30% of all infliximab discontinuations for AEs). Other common reasons for drug discontinuation for all three agents included infections, gastrointestinal disorders including nausea, vomiting and diarrhoea, and nervous system disorders, particularly headache. There was a tendency towards shorter persistence with treatment for infliximab when compared with the other two anti-TNF agents, as shown in Table 2.

**Table 3:** Patient withdrawal due to adverse events: first course of anti-TNF therapy

Adverse event leading to withdrawal (MedDRA system organ classification)	Etanercept (n = 316)	Infliximab (n = 162)	Adalimumab (n = 88)
Immune system disorders^a^	2 (0.6)	12 (7.4)	1 (1.1)
General disorders and administration site conditions^b^	2 (0.6)	2 (1.2)	0 (0.0)
Infections and infestations	15 (4.7)	3 (1.9)	3 (3.4)
Gastrointestinal disorders	4 (1.3)	4 (2.5)	1 (1.1)
Hepatobiliary disorders	1 (0.3)	2 (1.2)	0 (0.0)
Respiratory, thoracic and mediastinal disorders	1 (0.3)	0 (0.0)	0 (0.0)
Renal and urinary disorders	1 (0.3)	1 (0.6)	0 (0.0)
Cardiac disorders	1 (0.3)	3 (1.9)	0 (0.0)
Blood and lymphatic system disorders	2 (0.6)	1 (0.6)	2 (2.3)
Nervous system disorders	3 (0.9)	5 (3.1)	1 (1.1)
Skin and subcutaneous tissue disorders	3 (0.9)	3 (1.9)	3 (3.4)
Metabolism and nutrition disorders	0 (0.0)	0 (0.0)	1 (1.1)
Psychiatric disorders	1 (0.3)	0 (0.0)	0 (0.0)
Neoplasms benign, malignant and unspecified (including cysts and polyps)	3 (0.9)	2 (1.2)	1 (1.1)

Total	39 (12.3)	38 (23.5)	13 (14.8)

Data presented as n (%). ^a^Includes drug hypersensitivity. ^b^Includes one death of unknown cause.

### Predictors of persistence to the first anti-TNF therapy

Table 4 presents the results from the univariate and multivariate analyses examining predictors of drug overall discontinuation, discontinuation due to inefficacy and discontinuation due to AEs. For overall discontinuation, the multivariate model showed that being female (HR = 1.3, 95% CI = 1.0 to 1.7), having another baseline co-morbidity (HR = 1.5, 95% CI = 1.1 to 2.0) and using infliximab rather than etanercept (HR = 2.8, 95% CI = 2.1 to 3.7) were associated with significantly higher drug discontinuation rates.

**Table 4:** Univariate and multivariate Cox proportional hazard analysis for drug discontinuation due to inefficacy and adverse events

Variable	Overall withdrawal		Withdrawal due to inefficacy		Withdrawal due to adverse events	
			
	Univariate analysis	Multivariate analysis	Univariate analysis	Multivariate analysis	Univariate analysis	Multivariate analysis
Demographic						
Age at start of therapy (years)	0.99 (0.98 to 1.00)	0.99 (0.98 to 1.00)	0.99 (0.97 to 1.01)	0.99 (0.96 to 1.01)	0.98 (0.95 to 1.01)	0.96 (0.93 to 1.00)
Female	1.38* (1.12 to 1.70)	1.29* (1.01 to 1.65)	1.48 (0.92 to 2.37)	1.45 (0.82 to 2.64)	1.64 (0.87 to 3.09)	1.58 (0.78 to 3.22)
Smoking (yes/no)	1.36* (1.09 to 1.68)	1.22 (0.96 to 1.55)	1.70* (1.03 to 2.81)	1.51 (0.87 to 2.64)	1.10 (0.59 to 2.07)	0.97 (0.49 to 1.89)
Co-morbidity^a ^(yes/no)	1.77* (1.39 to 2.25)	1.49* (1.13 to 1.96)	1.64 (0.97 to 2.80)	1.17 (0.64 to 2.12)	2.68* (1.23 to 5.85)	2.67* (1.16 to 6.15)
Start year of therapy^b^						
2003	0.79 (0.56 to 1.13)	0.95 (0.98 to 2.21)	0.84 (0.37 to 1.95)	0.99 (0.73 to 5.44)	1.25 (0.42 to 3.72)	0.92 (0.70 to 3.04)
2004	0.65* (0.46 to 0.93)	0.93 (0.87 to 2.03)	0.71 (0.31 to 1.64)	0.81 (0.64 to 5.12)	0.70 (0.22 to 2.23)	0.96 (0.44 to 5.49)
2005	0.52* (0.34 to 0.80)	0.93 (0.74 to 2.19)	0.68 (0.26 to 1.79)	0.92 (0.61 to 7.45)	0.52 (0.13 to 2.19)	0.95 (0.28 to 7.55)
2006	0.43 (0.18 to 1.03)	0.94 (0.73 to 2.12)	0.99 (0.19 to 4.95)	0.94 (0.62 to 6.64)	0.99 (0.99 to 1.00)	0.93 (0.28 to 5.65)
Disease						
Baseline HAQ	1.16* (1.03 to 1.29)	1.08 (0.95 to 1.22)	1.26* (1.02 to 1.55)	1.20 (0.95 to 1.49)	1.16 (0.83 to 1.62)	1.06 (0.71 to 1.59)
Disease duration (years)	0.99 (0.98 to 1.00)	0.99 (0.98 to 1.01)	0.98 (0.95 to 1.00)	0.99 (0.97 to 1.03)	0.99 (0.96 to 1.03)	0.99 (0.95 to 1.03)
Baseline DAS-28	0.93 (0.85 to 1.01)	0.88 (0.79 to 1.16)	0.88 (0.73 to 1.07)	0.83 (0.66 to 1.04)	0.88 (0.69 to 1.12)	0.89 (0.66 to 1.57)
Inflammation^c^	0.93 (0.74 to 1.15)	--	0.72 (0.45 to 1.17)	--	1.18 (0.60 to 2.29)	--
Tender joint counts	1.00 (0.99 to 1.01)	--	0.99 (0.97 to 1.03)	--	1.00 (0.96 to 1.04)	--
Swollen joint counts	0.97* (0.96 to 0.99)	--	0.98 (0.94 to 1.02)	--	0.99 (0.94 to 1.04)	--
Therapeutic – concurrent use of:						
Methotrexate	0.89 (0.72 to 1.09)	0.64 (0.49 to 1.12)	1.03 (0.64 to 1.67)	0.65 (0.36 to 1.18)	1.06 (0.57 to 1.99)	0.67 (0.32 to 1.39)
Sulfasalazine	0.49 (0.29 to 1.15)	0.30 (0.13 to 1.19)	0.40 (0.10 to 1.64)	0.32 (0.04 to 2.36)	0.68 (0.16 to 2.81)	1.54 (0.34 to 6.92)
Steroids	1.22 (0.96 to 1.57)	1.06 (0.81 to 1.38)	1.14 (0.65 to 2.01)	0.91 (0.49 to 1.71)	1.89 (0.98 to 3.66)	1.92 (0.95 to 3.89)
Biological therapy^d^						
Infliximab	2.30* (1.85 to 2.87)	2.80* (2.12 to 3.70)	2.62* (1.59 to 4.34)	3.77* (1.96 to 7.24)	2.42* (1.26 to 4.68)	3.12* (1.41 to 6.89)
Adalimumab	1.11 (0.78 to 1.59)	1.00 (0.67 to 1.51)	1.81 (0.89 to 3.65)	1.62 (0.70 to 3.78)	1.11 (0.37 to 3.33)	0.74 (0.21 to 2.66)

Drug discontinuation due to inefficacy and adverse events. Data presented as hazard ratio (95% confidence interval). DAS-28, 28-joint disease activity score; HAQ, Health assessment questionnaire. ^a^Includes any of hypertension, angina, ischaemic heart disease, stroke, pulmonary fibrosis, asthma, chronic obstructive pulmonary disease, diabetes, thyroid disease, peptic ulcers, hepatic disease, renal disease, demyelinating disease, epilepsy, depression, tuberculosis, cancer. ^b^Reference category, 2002. ^c^Inflammation (C-reactive protein >20 mg/l or erythrocyte sedimentation rate >28 mm/hour). ^d^Reference category, etanercept. * *P *< 0.05.

For discontinuation due to inefficacy, the univariate analyses suggested that patients who smoked (HR = 1.7, 95% CI = 1.0 to 2.8), who had higher baseline HAQ scores (HR = 1.3, 95% CI = 1.0 to 1.6) or who were receiving infliximab (HR = 2.6, 95% CI = 1.6 to 4.3) were more likely to discontinue treatment due to inefficacy. In the multivariate analysis in which we simultaneously controlled for all of the potential explanatory variables, only treatment with infliximab rather than etanercept was found to be significantly associated with drug discontinuation for inefficacy (adjusted HR = 3.8, 95% CI = 2.0 to 7.3), although there was also a nonstatistically significant trend towards better survival in patients receiving concomitant disease-modifying anti-rheumatic drug therapy and in those with higher baseline disease activity.

For drug discontinuation due to AEs, the presence of baseline co-morbidities (HR = 2.7, 95% CI = 1.2 to 6.2) and the use of infliximab rather than etanercept (HR = 3.1, 95% CI = 1.4 to 6.2) were associated with significantly higher drug discontinuation rates. There were no significant associations between any of the other potential predictors.

### Persistence with the second anti-TNF therapy

A total of 178 patients received a second course of therapy with an alternative anti-TNF drug. Not surprisingly, the mean (standard deviation) length of observation was shorter for the second course of anti-TNF therapy (1.3 (0.8) person-years) compared with that of the first course (2.3 (0.9) person-years). Drug discontinuation rates in those patients who switched treatment to a second anti-TNF therapy are presented in Table 2. In general, persistence with the second course of therapy was lower than with the first course, although patient numbers were too small to study whether the reason for stopping the first course of anti-TNF could predict outcome on the second agent or to look at differences between the anti-TNF agents.

## Discussion

The present observational study, reflecting clinical practice in the UK, has shown that persistence with anti-TNF agents in PsA is good, with an estimated 1-year drug survival of 82%. Over a mean follow-up period of 2.3 person-years, 30.8% of patients discontinued their first anti-TNF treatment, mainly because of lack of efficacy (12.4%) or AEs (11.4%). The survivor function on second anti-TNF for switchers was 74% at 12 months.

Previous studies examining persistence with biological therapies in patients with PsA have only reported on persistence with the first anti-TNF treatment. An earlier study from the Spanish BIOBADASER register found that 88% of patients with PsA (n = 570) continued with their first anti-TNF drug for 12 months, compared with 83% of RA patients (n = 4,006) [20]. More recently, a Norwegian study found that 77.3% of PsA patients (n = 172) persisted with their anti-TNF for 12 months, compared with 65.4% of RA patients (n = 847) [21]. Our results would support the notion that patients with PsA have higher persistence rates with anti-TNF therapies than RA patients; an earlier analysis of RA patients (n = 6,739) registered with the BSRBR found that 65% of the observed RA cohort persisted with their primary anti-TNF therapy over a mean length of follow-up of 15 months [22]. Similarly, a French single-centre study found that 64% of rheumatic patients (n = 770) had not interrupted their anti-TNF therapies for 12 months [23].

To our knowledge, three other studies have formally explored possible predictors of drug discontinuation in patients with PsA treated with anti-TNF therapies. Kristensen and colleagues suggested that concomitant use of methotrexate and high C-reactive protein levels were associated with treatment continuation in a study of 261 PsA patients using anti-TNF drugs [24]. Gomez-Reino and colleagues reported that drug discontinuation was predicted by older age in 448 PsA patients [25], and Heiberg and colleagues found that higher baseline disease activity and female sex were associated with treatment continuation in 172 patients with PsA [21]. In our larger study, we found a trend towards a higher overall discontinuation rate in females. We also found a trend towards better drug survival in those with higher baseline disease activity; around 65% of our patient cohort had raised inflammatory markers and 53% were receiving methotrexate. Furthermore, the presence of co-morbidities was identified as a predictor of higher withdrawal rates for adverse events.

Data from the BSRBR reflect routine clinical practice in one of the largest prospective cohorts of patients with PsA. The register is therefore well suited for an investigation of drug persistence and switching between anti-TNF therapies. There are potential limitations to the present study, however, which should be considered when interpreting the findings. First, treatment decisions were not randomised but were left to the discretion of the treating physician, based on clinical opinion as to whether a patient's treatment had failed and the initial anti-TNF therapy should be discontinued. Second, as the BSRBR was originally developed as a RA register, there are certain aspects of the patient's PsA that were not captured – such as the pattern of joint involvement (that is, axial or peripheral) and the Psoriasis Area and Severity Index scores, which may also have influenced the physician's decision on choice of anti-TNF agent as well as judgements about lack of efficacy.

A full set of domains to assess improvements in PsA patients would ideally include outcomes that measure skin involvement, dactylitis, enthesitis, spine involvement and radiological outcomes. For feasibility reasons, however, the short form of the Disease Activity Score (the DAS-28) was used, which has been shown to be discriminant between anti-TNF drugs and methotrexate in PsA patients [26]. Physical function was also assessed using the HAQ, which was originally developed for RA [27] but has recently been validated for PsA [28]. The HAQ has been used extensively in clinical trials and observational studies [29,30], and has been shown to be adequately sensitive to peripheral disease improvements after anti-TNF therapies in PsA [3,7].

The present observational study found that, for patients starting anti-TNF therapy between 2002 and 2006, there was better persistence with etanercept when compared with infliximab, which is in accordance with data published earlier from the Swedish [24] and the Spanish [25] biologic registers. There are several possible reasons to explain why the observed drug persistence with infliximab was less than that observed for etanercept that should be taken into consideration before any formal conclusions are drawn.

Firstly, infliximab was the first anti-TNF drug to be marketed and, during the early years of this study, more patients were receiving infliximab as their first anti-TNF therapy, despite the drug not being licensed for use in PsA. These early patients were likely to be those with the most severe disease, and as a consequence they may have had a different response to those recruited later; the potential risk of channelling bias can therefore not be discounted [31]. Adjusting for calendar year in our multivariate model, however, did not affect these results. There was also a worldwide shortage of etanercept during the early years, and adalimumab was marketed later than infliximab and etanercept. As other drugs became available, patients may have decided to switch therapy for reasons in addition to those listed. An additional analysis that restricted the cohort to those patients recruited after 2003, however, found similar results.

Furthermore, and perhaps most importantly, only 22% of patients treated with infliximab were receiving the licensed dose for PsA (5 mg/kg), with the remaining 78% receiving the dose of 3 mg/kg recommended for use in RA. This is probably a result of the drug not receiving its license for use in PsA until 2004 and national guidelines not being issued until 2005, and thus most physicians may have applied the same dosing regimen used for RA to these patients. In keeping with this, 73% of patients with PsA included in the present study received infliximab before the date the drug was licensed for this specific indication. All of these reasons may explain the observed differences in drug survival, and therefore no definite conclusions about differential drug efficacy should be made from the present analysis.

## Conclusions

The present study has provided important insights into persistence rates with anti-TNF therapies in a large unselected population of patients with PsA. We have demonstrated that persistence in patients with PsA is at least as good as in those with RA, and that the main predictor of stopping for AEs is the presence of baseline co-morbidities. Our results have also shown that the survivor function in those patients receiving a second anti-TNF therapy was 74% at 12 months. Further studies are needed to explore the clinical effectiveness and cost-effectiveness of sequential use of different anti-TNF therapies in patients with PsA.

## Abbreviations

AE: adverse event; BSRBR: British Society for Rheumatology Biologics Register; CI: confidence interval; DAS-28: 28-joint Disease Activity Score; HAQ: Health Assessment Questionnaire; HR: hazard ratio; IFN: interferon; IL: interleukin; PsA: psoriatic arthritis; RA: rheumatoid arthritis; TNF: tumour necrosis factor.

## Competing interests

The British Society for Rheumatology commissioned the Biologics Register (BSRBR) as a UK-wide national project to investigate the safety of biologic agents in routine medical practice. DPMS and KLH are principal investigators on the BSRBR. The British Society for Rheumatology receives restricted income from UK pharmaceutical companies, presently Abbott Laboratories, Amgen, Schering Plough and Wyeth Pharmaceuticals. This income finances a wholly separate contract between the British Society for Rheumatology and the University of Manchester, who provide and run the BSRBR data collection, management and analysis services. The principal investigators and their team have full academic freedom and are able to work independently of pharmaceutical industry influence. All decisions concerning analyses, interpretation and publication are made autonomously of any industrial contribution. Members of the Manchester team, British Society for Rheumatology trustees, committee members and staff complete an annual declaration in relation to conflicts of interest.

## Authors' contributions

All authors were involved in protocol development, data analysis, interpretation of the findings and preparation of the final manuscript. AAS also carried out data extraction and prepared the first draft of the manuscript. DPMS and KLH are the principal investigators on the BSRBR, and KDW is the study coordinator.
